# A small pancreatic hamartoma with an obstruction of the main pancreatic duct and avid FDG uptake mimicking a malignant pancreatic tumor: a systematic case review

**DOI:** 10.1186/s12876-017-0704-8

**Published:** 2017-12-06

**Authors:** Hiroaki Nagano, Masayuki Nakajo, Yoshihiko Fukukura, Yoriko Kajiya, Atsushi Tani, Sadao Tanaka, Mari Toyota, Toru Niihara, Masaki Kitazono, Toyokuni Suenaga, Takashi Yoshiura

**Affiliations:** 1 Departments of Radiology, Nanpuh Hospital, 14-3 Nagata, Kagoshima, 892-8512 Japan; 2 Departments of Pathology, Nanpuh Hospital, 14-3 Nagata, Kagoshima, 892-8512 Japan; 3 Departments of Gastroenterology, Nanpuh Hospital, 14-3 Nagata, Kagoshima, 892-8512 Japan; 4 Departments of Surgery, Nanpuh Hospital, 14-3 Nagata, Kagoshima, 892-8512 Japan; 5 Department of Radiology, Kagoshima University Graduate School of Medical and Dental Sciences, 8-35-1 Sakuragaoka, Kagoshima-shi, Kagoshima, 890-8544 Japan

**Keywords:** Hamartoma, Pancreas, CT, MRI, FDG, PET/CT

## Abstract

**Background:**

Pancreatic hamartomas are extremely rare and may be misdiagnosed as malignant tumors. We report herein a case of a small, solid-type pancreatic hamartoma.

**Case presentation:**

A 72-year-old female was incidentally detected pancreatic lesion by ultrasonography. Computed tomography and magnetic resonance imaging revealed a 2.0-cm solid lesion. The main pancreatic duct (MPD) was obstructed by the lesion in the head of the pancreas, and the upstream MPD was dilated. ^18^F-fluorodeoxyglucose (FDG) accumulated avidly in the lesion and increased in FDG intensity from the early to the delayed images. The histopathological studies confirmed the diagnosis of pancreatic hamartoma. Immunohistochemically, the cell membrane of the accessory glands and ducts showed homogeneous expression of glucose transporter type I and hexokinase II.

**Conclusion:**

Pancreatic hamartomas causing dilatation of the MPD are extremely rare, and this appears to be the first case of a hamartoma to take up FDG avidly. It was a rare occurrence and should be noted that pancreatic hamartomas can cause an obstruction of the MPD and show avid FDG uptake, thereby mimicking malignant pancreatic tumors.

## Background

Hamartomas are benign, tumor-like nodules composed of an overgrowth of mature cells and tissues that normally occur in the affected tissue, but often with one predominant element. Pancreatic hamartomas are composed of three disarranged cellular components in varying proportions: acinar, islet, and ductal cells [1]. These hamartomas are extremely rare and may be misdiagnosed as malignant tumors. Herein, we report a case of pancreatic hamartoma resembling a malignant pancreatic tumor.

## Case presentation

A 72-year-old asymptomatic woman was referred to our hospital for examination of a pancreatic lesion detected on abdominal ultrasonography during a health screening. Her past medical history and physical examination were unremarkable. On laboratory testing, the serum levels of amylase, bilirubin, carcinoembryonic antigen, and carbohydrate antigen 19-9 were within normal limits.

**Fig. 1:**
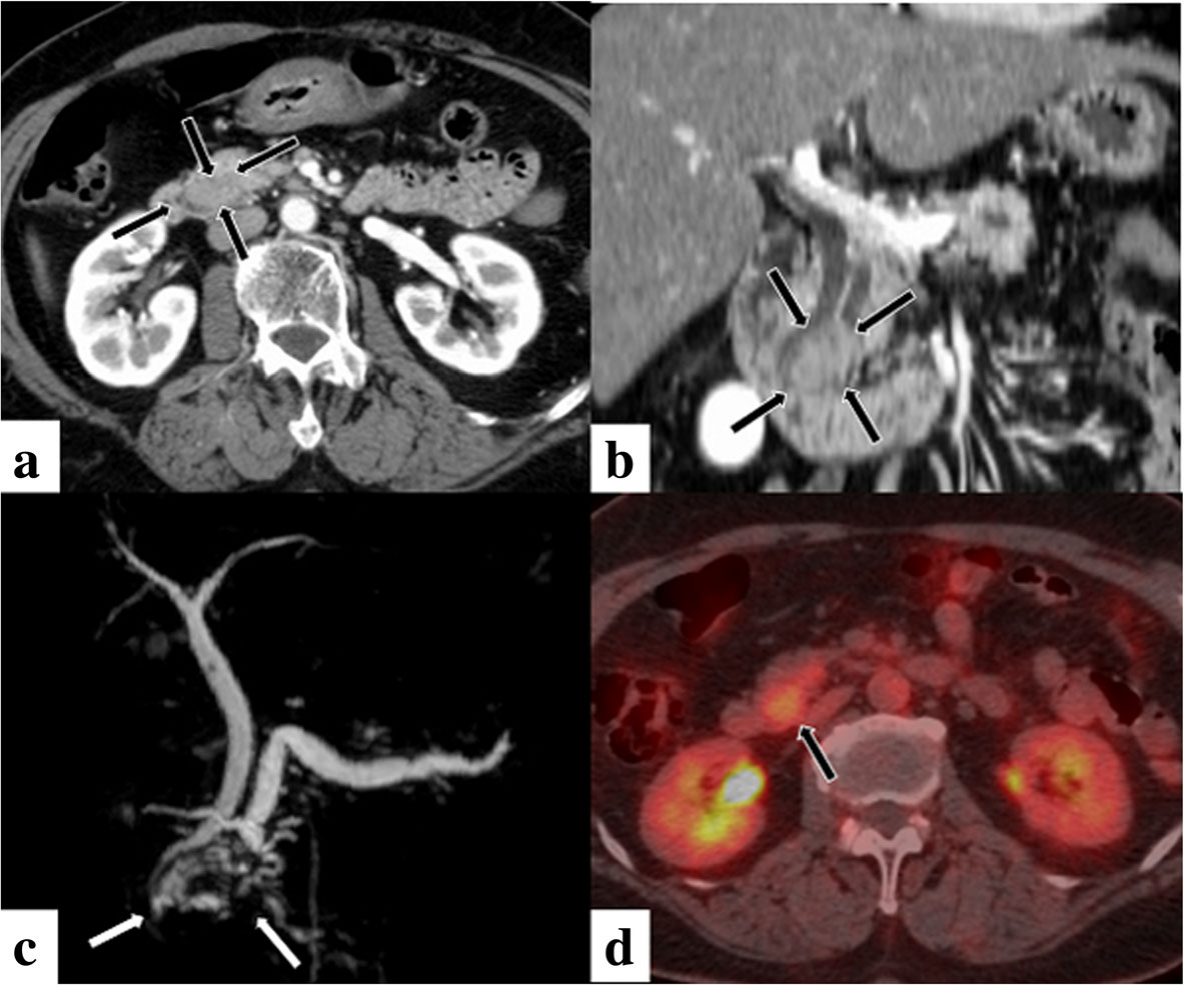
Contrast-enhanced CT axial (**a**) and coronal (**b**) images demonstrates a 2.0 cm, non-deforming mildly hypoattenuating mass in the pancreatic head (arrows) associated with obstruction of the pancreatic duct. MRCP (**c**): Confirms the dilatation of the pancreatic duct (8 mm in diameter) in the body of the pancreas. Note the associated dilatation of the Santorini duct (small arrows). FDG-PET/CT (**d**): The pancreatic lesion (arrow) shows avid ^18^F-fluorodeoxyglucose (FDG) uptake on the early image. The lesion increases in visual intensity on the delayed image on FDG-PET/CT

Abdominal ultrasonography revealed a heterogeneous, hypoechoic mass in the pancreatic head. Dynamic computed tomography (CT) demonstrated a non-deforming mass in the pancreatic head measuring 2.0 cm in maximum diameter and a dilated upstream of the main pancreatic duct (MPD) (Fig. 1a and b). The lesion showed isoattenuation on unenhanced CT, mild hypoattenuation during the pancreatic parenchymal phase, and isoattenuation during the portal venous and delayed phases relative to the surrounding pancreatic parenchyma. On magnetic resonance imaging (MRI), the lesion showed heterogeneous low-signal intensity on T1-weighted imaging and mild high-signal intensity on T2-weighted imaging compared to the surrounding pancreatic parenchyma. On diffusion-weighted imaging, the lesion showed mild high-signal intensity with an apparent diffusion coefficient value of 1.4 × 10^-3^ mm^2^/s. On MR cholangiopancreatography (MRCP) (Fig. 1c), the lesion obstructed the MPD in the head of the pancreas, and the upstream pancreatic duct was smoothly dilated (8 mm in diameter). Endoscopic retrograde cholangiopancreatography (ERCP) showed a stricture of the MPD with a dilated upstream. Endoscopic ultrasound (EUS) showed a 24 mm mural hyperechoic mass in the MPD. On positron emission tomography/CT (PET/CT), the pancreatic lesion showed avid ^18^F-fluorodeoxyglucose (FDG) uptake with a maximum standardized uptake value (SUVmax) of 3.6 at the early imaging (1 h after intravenous FDG injection) (Fig. 1d), which increased to 5.0 at the delayed imaging (2 h after intravenous FDG injection).

**Fig. 2:**
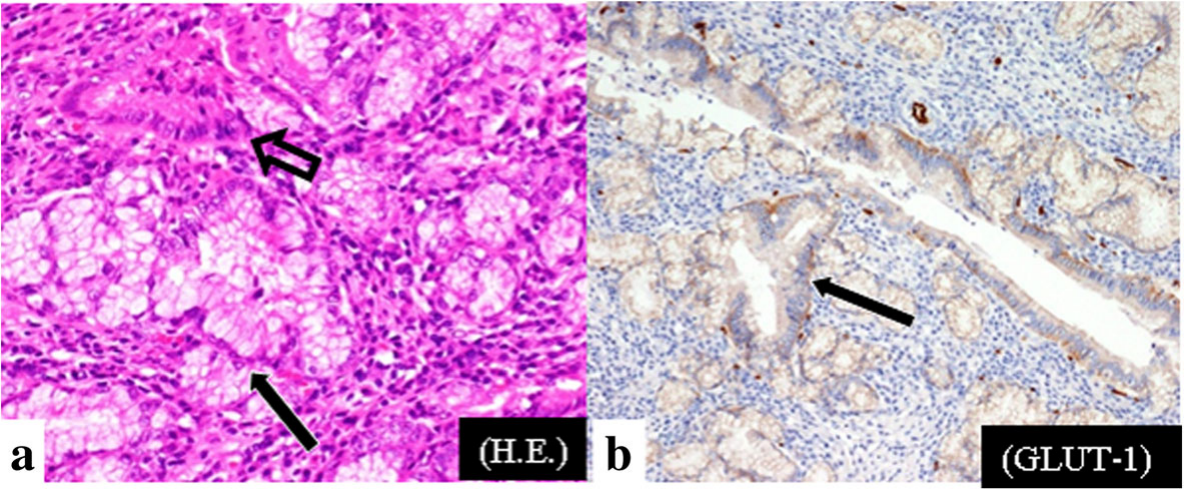
Macroscopic and immunohistochemical findings. **a**: The lesion is composed of accessory glands (closed arrows) and ducts lined by cuboidal to flattened epithelium, without atypia (open arrows). **b**: On immunohistochemistry, the cell membrane of the accessory glands and ducts show homogeneous expression of glucose transporter type I. The expressions are especially strong along the basilar membrane of the accessory glands (closed arrows)

The patient then underwent a subtotal pyloric-preserving pancreaticoduodenectomy. Macroscopically, a well-demarcated, homogeneous, white-to-yellow-colored solid lesion measuring 2.0 cm was noted in the pancreas head. Microscopically, the solid lesion was composed of accessory glands and ducts, lined by cuboidal to flattened epithelium without atypia (Fig. 2a). The accessory glands and ducts were embedded in a fibrotic stroma, and the islets of Langerhans were not evident within the lesion. Lymphocyte and follicle formation infiltrated diffusely around the MPD. Immunohistochemically, the ductal cells were positive for epithelial markers, but negative for synaptophysin and chromogranin A. The cell membrane of the accessory glands and ducts showed homogeneous expression of glucose transporter type I (GLUT-1) (Fig. 2b) and hexokinase II (HK-II). The expressions were especially strong along the basilar membrane of the accessory glands. The pathological diagnosis was solid-type pancreatic hamartoma. After surgical resection, the patient received follow-up CT examinations every three to 6 months and there has been no recurrence for 36 months.

## Discussion

Pancreatic hamartomas are extremely rare, and their radiologic features have not been fully evaluated. To the best of our knowledge, there have been only 30 previous reports of pancreatic hamartomas in the literature, and there are very few reports regarding their imaging appearance [1–20]. Age at presentation ranged from 34 weeks to 78 years (mean, 48.6 years). Both sexes were equally affected, with a male-to-female ratio of 1:0.82. Most patients were either asymptomatic or they exhibited symptoms related to local mass effect, including abdominal pain, weight loss, and a palpable mass; and only one patient had jaundice, caused by obstruction of the common bile duct [17]. Pancreatic hamartomas were reported to arise from all parts of the pancreas, although the pancreatic head was the most common site (64.5%, 20/31). Their size ranged from 1.0–14.0 cm (mean, 4.5 cm).

Morphologically, there are two types of pancreatic hamartoma: solid, and solid and cystic [2, 3]. Nine of the previously reported cases of pancreatic hamartoma seemed to fit the criteria of solid type; however, only three reports included the radiological appearance [1, 16, 17]. One showed that the MPD was compressed by the hamartoma located in the pancreatic body, and the upstream was dilated [16]. The remaining two cases had no stricture or dilatation of the MPD [1, 17]. On enhanced CT, one case showed a hyperattenuating hamartoma during the portal venous phase [17], and the other two cases demonstrated delayed enhancement on contrast-enhanced CT or MRI [1, 16]. In the present case, a pancreatic hamartoma measuring 2.0 cm in diameter was located in the head of the pancreas and caused an obstruction of the MPD, with a dilated upstream on CT, MRCP, and ERCP. Contrast-enhanced CT demonstrated an ill-defined lesion with mild hypoattenuation during the pancreatic parenchymal phase and isoattenuation during the portal venous and delayed phases.

Pancreatic ductal adenocarcinomas may present as a non-deforming iso- or hypoattenuation mass during the pancreatic parenchymal to portal venous phase on enhanced CT [21], and have an obstruction of the MPD with upstream ductal dilatation on CT or MRI [21, 22]. Pancreatic neuroendocrine tumors usually show hypervascular patterns during the arterial to the portal venous phase. However, high-grade pancreatic neuroendocrine tumors tend to show hypovascular patterns and an interruption of the MPD with upstream ductal dilatation [23–25]. Therefore, in the present case, it was difficult to differentiate pancreatic hamartoma from ductal adenocarcinoma, neuroendocrine tumor, or pancreatic metastases.

On FDG-PET, no avid FDG uptake was noted in the three previously reported cases of pancreatic hamartoma [9, 14, 15]. Ours appears to be the first case of a pancreatic hamartoma to show avid FDG uptake, which may be related to the GLUT-1 and HK-II expression of the accessory glands and ducts. Yoshioka et al. [26] reported that an FDG SUVmax of 2.5 would be justified as a cut-off value to differentiate between malignant and benign pancreatic tumors. Kawada et al. [27] reported that the FDG SUVmax increased from the early to the delayed images in 89% (39/44) of the small malignant pancreatic tumors (< 25 mm). In the present case, the FDG SUVmax was 3.6 at the early phase and increased to 5.0 at the delayed phase, in concordance with malignant pancreatic tumors. In our case, therefore, it was difficult to differentiate from malignant tumors based on US, CT, MRI, and FDG-PET/CT findings. Pancreatic hamartoma is benign entity, and does not require surgical treatment. EUS-Fine-Needle-Aspiration might have been effective for definitive diagnosis if we had performed it.

## Conclusion

Pancreatic hamartomas causing dilatation of the MPD were extremely rare, and showed no avid FDG uptake in the previously reported cases. We described a rare case of a small, solid-type pancreatic hamartoma that showed an obstruction of the main pancreatic duct and avid FDG uptake. Radiologists should be aware that pancreatic hamartomas may be associated with these findings.

## Abbreviations

CT: Computed tomography
ERCP: Endoscopic retrograde cholangiopancreatography
EUS: Endoscopic ultrasound
FDG: ^18^F-fluorodeoxyglucose
GLUT-1: Glucose transporter type I
HE: Hematoxylin-eosin staining
HK-II: Hexokinase II
MPD: Main pancreatic duct
MRCP: MR cholangiopancreatography
MRI: Magnetic resonance imaging
PET/CT: Positron emission tomography/CT
SUVmax: Maximum standardized uptake value
